# Impact of routine prophylaxis with monoclonal antibodies and maternal immunisation to prevent respiratory syncytial virus hospitalisations, Lombardy region, Italy, 2024/25 season

**DOI:** 10.2807/1560-7917.ES.2025.30.14.2400637

**Published:** 2025-04-10

**Authors:** Francesco Menegale, Luigi Vezzosi, Marcello Tirani, Simona Scarioni, Stefano Odelli, Federica Morani, Catia Borriello, Elena Pariani, Ilaria Dorigatti, Danilo Cereda, Stefano Merler, Piero Poletti

**Affiliations:** 1 Center for Health Emergencies, Fondazione Bruno Kessler, Trento, Italy; 2 Department of Mathematics, University of Trento, Trento, Italy; 3 Lombardy Region Welfare General Directorate, Milan, Italy; 4 Department of Biomedical Sciences for Health, University of Milan, Milan, Italy; 5 MRC Centre for Global Infectious Disease Analysis, School of Public Health, Imperial College London, London, United Kingdom

**Keywords:** respiratory syncytial virus, monoclonal antibodies, respiratory syncytial virus vaccines, transmission model, immunization programs, maternally-acquired immunity

## Abstract

**Background:**

Respiratory syncytial virus (RSV) is a leading cause of hospitalisation in children worldwide. Recent regulatory approval of monoclonal antibody (mAb) nirsevimab for infants and the RSVpreF vaccine for pregnant women offers promising approaches to mitigate RSV-associated morbidity.

**Aim:**

To evaluate potential impacts of routine prophylactic campaigns (mAbs targeting infants or maternal vaccination) introduced in the 2024/25 season on hospitalisations from RSV lower respiratory tract infections in Lombardy, Italy.

**Methods:**

We used a catalytic model informed by data from pre-COVID-19 pandemic (before 2020) and post-pandemic periods (until 2022) to quantify the number of cases and hospitalisations that could be averted by seasonal nirsevimab administration to infants and RSVpreF maternal vaccination, considering changes in susceptibility caused by reduced RSV circulation during the pandemic.

**Results:**

As a marked proportion of RSV hospitalisations occurs in infants aged ≤ 1 year, seasonal mAb administration to 80% of newborns (uptake levels observed in Spain) was estimated to avert 50.2% (95% CI: 43.5–55.8) of hospitalisations in the total population. Coverage levels close to those observed for childhood vaccines (95%) could result in an additional average 18% reduction in hospitalisations. Vaccination of 65% of pregnant women, resembling the diphtheria–tetanus–pertussis vaccine coverage in Lombardy for this population, was estimated to avert 30.5% (95% CI: 19.6–39.7) of hospitalisations. At influenza vaccine coverage (12%), less than 8% of hospitalisations could be averted by maternal immunisation.

**Conclusion:**

Routine nirsevimab administration to infants demonstrates clear potential to reduce RSV-associated hospitalisations. Maternal immunisation can help in achieving high protection in at-risk populations.

**Key public health message**

**What did you want to address in this study and why?**

Respiratory syncytial virus (RSV) is a leading cause of respiratory infection in infants and young children. Newly developed maternal vaccines and monoclonal antibodies have great potential to reduce severe outcomes and hospitalisations caused by RSV infection. We aimed to evaluate the impact of routine use of these products to reduce the burden of RSV disease in Lombardy, Italy, for the 2024/25 season.

**What have we learnt from this study?**

Without interventions, most RSV-associated hospitalisations occur among infants under 1 year. Administering monoclonal antibodies to 80% of infants can reduce hospitalisations in the overall population by about 50%, averting more than 1,400 hospital admissions from lower respiratory tract infections annually in Lombardy. Vaccination of 65% of pregnant women can prevent around 30% of RSV hospitalisations in the overall population, equating to ~ 860 hospitalisations per year.

**What are the implications of your findings for public health?**

The introduction of immunisation programmes based on monoclonal antibodies and maternal vaccination can markedly reduce the hospital burden associated with RSV. Administration of monoclonal antibodies to infants is likely to be more effective than maternal vaccination, as it is expected to have higher uptake and efficacy.

## Introduction

Respiratory syncytial virus (RSV) is a leading cause of acute lower respiratory tract infections (LRTI) in children under 5 years of age worldwide [1,2]. Infants are at higher risk of developing severe disease, with around 1.4 million RSV-associated hospitalisations and 45,700 RSV-attributable deaths estimated globally in 2019 among children aged 0–6 months [2]. For over 2 decades, the only specific immunoprophylaxis available was palivizumab, an expensive monoclonal antibody (mAb) with a short half-life (in the range of 18–21 days, thus requiring five doses over a winter season), and therefore administered exclusively to high-risk infants (aged < 1 year) in high-income countries [3]. The recent approval by the European and American regulatory agencies [4-7] of nirsevimab (Beyfortus, Sanofi and AstraZeneca), a single-dose mAb with an extended half-life of ca 71 days [8], and of the RSVpreF vaccine (Abrysvo, Pfizer), a single-dose vaccine for use in pregnant women, has provided promising prophylactic strategies to reduce the disease burden of RSV in infants.

Nirsevimab administration is recommended for infants born during or entering their first RSV season, and for children up to 24 months of age who are at increased risk of severe RSV disease and are entering their second RSV season [4,6,9]. Nirsevimab administration has been shown to provide protection against medically attended LRTI throughout an infant’s first RSV season [10], without enhancing the risk of severe disease in the subsequent season [11]. Administration of RSVpreF vaccine is recommended to pregnant women between 32 and 36 completed gestational weeks [5,7]. Maternal vaccination leads to the passive immunisation of the newborns through transplacental antibody transfer, with protection against medically attended LRTI estimated to last at least 6 months [12,13]. 

We evaluated the impact of introducing seasonal immunoprophylactic campaigns starting in the 2024/25 season in the Lombardy region, which is home to ca 10 million inhabitants in northern Italy [14]. In this region, after 2 years of low RSV circulation, a concerning rise in hospitalisations was observed during the 2021/22 season, with more than 2,700 patients hospitalised with this viral infection, 78% of whom were younger than 1 year of age [15]. This rise occurred following the disruption of the typical seasonal RSV circulation caused by non-pharmaceutical interventions implemented between 2020 and 2021 to mitigate the COVID-19 pandemic. A recently published study [15] employed a catalytic model to analyse surveillance and hospitalisation data from Lombardy and quantified the effect of the COVID-19 restrictions on RSV circulation between 2020 and 2021. Building upon this study, our work evaluates the impact of alternative prophylaxis measures that could be implemented to reduce the burden of RSV, by means of a catalytic approach. Catalytic models, which link the number of infected individuals directly to the rate of new infections, require fewer assumptions about temporal changes in the transmission dynamics and in the viral circulation than compartmental models, making them particularly suited for estimating attack rates during atypical seasons and epidemic waves, such as those observed during the COVID-19 pandemic. Here, we extend this modelling framework to assess the potential impact of using mAb administration in infants, vaccination during pregnancy, or a combination of the two strategies on reducing the number of hospitalisations associated with RSV infections in Lombardy for the 2024/25 season, both in the target group (infants) and in the overall population.

## Methods

### Model structure and assumptions

We extended a published catalytic model for RSV [15] to evaluate the impact of introducing routine mAb administration in infancy and/or routine maternal vaccination on the incidence of medically attended cases and hospitalisations caused by RSV-associated LRTI in Lombardy. We assume that these new prophylactic interventions (alone or combined) are introduced before the 2024/25 RSV season targeting infants. Similarly to previous studies [15-17], we assume that, in any season, all individuals are exposed to the risk of RSV infection, but that individuals can only get infected once per season. In Lombardy, the RSV season usually starts in November and ends in April of the successive year [15]. Following their primary infection, individuals are assumed to experience a reduced risk of re-infections in the subsequent seasons (23% lower risk of infection [15,16]). The model considers a population stratified by age (namely, 100 age groups from 0 to 99 years, plus one age group for individuals aged 100 years or older). The population is assumed to be of constant size and with constant age distribution.

Mimicking the policies implemented in the clinical trials [12,18,19], we assume that mAbs are administered to infants shortly before the start of the RSV season and that the vaccine is administered to pregnant women who give birth to infants entering their first RSV season in 2024. Both mAbs and vaccine provide partial protection against RSV infection during the first RSV season experienced after administration, with a reduction in the infection risk ranging from 62% to 85% [18] and from 30% to 75% [13], respectively. Available evidence [11] suggests that nirsevimab is not associated with an increased incidence or severity of RSV LRTI cases during the second year of life in infants who received nirsevimab before their first RSV season. In our analysis, we assumed that both products do not increase the risk of enhanced disease in the subsequent RSV seasons. In our model, as suggested by the guidelines provided by the Advisory Committee on Immunization Practices (ACIP), individuals can be immunised by either mAbs or vaccine, and the administration of both products to protect the same individual is not considered [13]. In our main analysis, coverage is assumed to be 80% for mAbs, similar to preliminary coverage levels observed in 2023 in Spain among non-high-risk individuals [20]. As for vaccination during pregnancy, we assume 65% coverage, similar to the uptake of the diphtheria-tetanus-pertussis (DTP) vaccine in Lombardy among pregnant women in 2023. Alternative coverage levels are explored in a sensitivity analysis.

Following the catalytic approach, we define the probability of primary infection among individuals aged less than 1 year (*a* = 0) in season *y* as:


$$P_{0,y}^{1}(\varepsilon )=\left(1-\varepsilon \right)\left(1-e^{-\lambda \left(0,y\right)}\right)\;(1)$$

where *ε *is equal to the efficacy of the immunoprophylaxis in reducing the risk of RSV infection for individuals who were targeted either by mAbs or vaccine, and equal to 0 for non-immunised individuals. *λ*(0*,y*) defines the force of infection of RSV in unprotected infants, representing the per-capita rate at which naive individuals are infected in season *y*, given that they had never experienced the infection in the past. In the absence of interventions (baseline scenario), the force of infection is modelled as age-dependent and proportional to the overall number of RSV cases reported to the regional healthcare system. When the impact of prophylaxis measures is explored, the model keeps into account the indirect effect of the considered intervention (‘mAbs’, ‘vaccine’, ‘combined’ scenarios) on the RSV circulation in the general population (see Supplementary section ‘Mathematical model’ for more details on the indirect effect of the interventions). The probability of primary infection for individuals of age *a* > 0 is:


$$P_{a,y}^{1}(\varepsilon )=\left(1-\;e^{-\lambda \left(a,y\right)}\right)\left(e^{-\sum_{i=1}^{a-1}\lambda \left(i,\;y-a+i\right)}\right)\left({1-P}_{0,y-a}^{1}(\varepsilon )\right)\;(2)$$

where the first term represents the probability of getting infected in season *y*, the second term the probability of remaining susceptible from the second year of life until season *y*, and the third term the probability of remaining susceptible after the first year of life, which depends on whether they received or not any prophylaxis in their first year of life. Similarly, the probability of a post-primary RSV infection for individuals of age *a* > 0 is:


$$P_{a,y}^{2}(\varepsilon )=\left(1-\;e^{-s∙\lambda \left(a,y\right)}\right)\left[{1-\;e}^{-\sum_{i=}^{a-1}\lambda \left(i,\;y-a+i\right)}∙\left({1-P}_{0,y-a}^{1}(\varepsilon )\right)\right]\;(3)$$

where the first term represents the probability of getting re-infected in season *y* with *s* = 0.77 accounting for the 23% reduced risk against re-infections and the second term the probability of having experienced a primary RSV infection in the past. Finally, the overall probability of RSV infection for each age and year *P_a,y_* is derived by summing the probability of primary and post-primary infection among individuals who were ever targeted by the immunising intervention and those who did not:


$$P_{a,y}\;=c_{mAbs}\left(y-a\right)\left(P_{a,y}^{1}\left(\varepsilon_{mAbs}\right)+P_{a,y}^{2}\left(\varepsilon_{mAbs}\right)\right)+c_{vax}\left(y-a\right)\left(P_{a,y}^{1}\left(\varepsilon_{vax}\right)+P_{a,y}^{2}\left(\varepsilon_{vax}\right)\right)\;(4)+\left(1-c_{mAbs}\left(y-a\right)-c_{vax}(y-a)\right)\left(P_{a,y}^{1}(0)+P_{a,y}^{2}(0)\right)$$

where efficacy values for mAbs (*ε_mAbs_*) and for vaccine (*ε_vax_*) were sampled from the estimates obtained in the clinical trials for the two products [13,18], and *c_mAbs_(y – a)* and *c_vax_(y – a)* denote the coverage levels in season *y – a* for the two immunoprophylaxes. For each season, the model provides estimates of the number of all infections (including infections undetected by the surveillance system) occurring in each age group. The age-specific and overall number of RSV cases reported to the surveillance system and hospitalisations caused by RSV are derived considering age-specific ascertainment and hospitalisation rates. 

### Model calibration

The model was parametrised to match both the overall and age-specific data on the number of cases and on hospitalisations caused by RSV circulation from 2018 to 2022, using a Markov Chain Monte Carlo (MCMC) Bayesian inferential framework [15]. Free model parameters included age- and season-specific transmission rates, and age-specific reporting and hospitalisation rates. Data employed for model calibration were provided by the Welfare General Directorate of the Lombardy Region and consisted of official records on the number and the age distribution of RSV-attributable cases with LRTI seeking medical care and RSV-attributable hospitalisations occurred in the entire region over the period 2018–22. Specifically, the number and age distribution of RSV-attributable hospitalisations were obtained by official hospital discharge records [15,21]. The number and age distribution of ‘RSV-attributable cases’ were derived by combining the size of the Lombardy population [22] with age-stratified records of outpatients seeking care for influenza-like illness (ILI) and the season-specific RSV test positivity rate, as collected within the Italian respiratory virus surveillance network (RespiVirNet, formerly InfluNet) [15,23]. Virological tests were conducted on a sub-sample of outpatients with ILI recorded through the influenza surveillance network. The model fit to these data are displayed in Supplementary Figures S2–S3. 

### Main analysis

The calibrated model is used to estimate the time-varying proportion of individuals naive to RSV infection before the start of the season, the age-specific incidence of RSV infections over time, and the number of averted cases and hospitalisations by mAb administration and/or maternal vaccination compared with a counterfactual scenario with no intervention in the 2024/25 season. In the absence of interventions, the force of infection and age-dependent transmission rates from the 2022/23 season onwards were assumed to be equal to those estimated for the last RSV season observed in Lombardy before the emergence of the COVID-19 pandemic (2018/19) [15]. The immunity profile of the population (i.e. the age-specific proportion of individuals who have never been exposed to primary RSV infection) is allowed to vary over the years and was initialised at the levels estimated for the 2021/22 season [15]. To emphasise the distinct age distribution of RSV cases and hospitalisations, as well as the indirect effects of infant prophylaxis on older age groups, model estimates are shown for the following age groups: 0–6 months old, 7–12 months old, 1–2 years old, 3–49 years old and ≥ 50 years old.

The model estimates age-specific RSV cases and hospitalisations separately for each season by analytically computing the probability of infection, incorporating the likelihood of having escaped infection in previous seasons. Consequently, in contrast with dynamical compartmental models, where uncertainty may increase over longer time horizons, uncertainty in future projections arises solely from the variability of model parameters and the estimated temporal changes in individuals’ infection histories. Different model outcomes presented in this paper were obtained considering 5,000 samplings drawn from the posterior distribution of free model parameters and ranges of efficacy values estimated for the two products in clinical trials. Resulting estimates are reported as mean values with 95% credible intervals (CI), reflecting the uncertainty associated with model parameters (see Supplementary Table S1 and [13,18] for the estimates of the model parameters). Further methodological details are reported in the Supplementary Material.

### Alternative scenarios and sensitivity analyses

In our main analysis, we assumed a mAb uptake based on early records concerning the nirsevimab campaigns conducted in Spain in 2023 [20] and a vaccine coverage resembling the vaccine uptake observed among pregnant women in 2023 for the diphtheria-tetanus-pertussis (DTP) vaccine in Lombardy. We explore alternative coverage scenarios by assuming that mAb coverage could either be markedly lower than the one observed in Spain, such as 70%, or reach higher uptake levels, as reported in some Spanish regions for infants born during the 2023/24 RSV season [24,25] and as recorded in Lombardy for other childhood diseases such as measles, such as 95%. As for maternal vaccination, we consider an optimistic scenario with a vaccine uptake equal to the baseline mAb coverage (80%) [20], and a pessimistic scenario assuming the uptake level recorded in Lombardy in 2022/23 for maternal vaccination against influenza (12%). Finally, we explore three scenarios combining maternal vaccination with mAb administration. We considered a pessimistic scenario, an optimistic scenario and an intermediate one, defined by the combination of lowest, central and highest values of coverage considered for single interventions assuming that mAb coverage applies to infants whose mothers did not receive vaccination during pregnancy. Considered scenarios can be found in the Table.

**Table:** Number and percentages of averted RSV cases and hospitalisations in the overall population following introduction of monoclonal antibody administration in infancy or vaccination during pregnancy under different scenarios, Lombardy, Italy, 2024/25 season (n = 9,965,046 inhabitants^a^)

Scenario^b^	Efficacy^c^		Coverage		Number of averted cases		Per cent averted cases		Number of averted hospitalisations		Per cent averted hospitalisations	
	mAb	Vaccine	mAbs	Vaccine								
					n	95% CI	%	95% CI	n	95% CI	%	95% CI
mAb	62–85%	n/a	80%	n/a	10,949	9,469–12,190	5.75	4.97–6.40	1,421	1,225–1,586	50.22	43.54–55.79
			70%		9,590	8,291–10,677	5.03	4.35–5.60	1,246	1,073–1,391	44.04	38.16–48.93
			95%		12,984	11,232–14,452	6.82	5.90–7.58	1,682	1,451–1,876	59.46	51.56–66.01
Vaccination	n/a	30–75%	n/a	65%	6,623	4,238–8,654	3.48	2.23–4.54	862	554–1,124	30.48	19.58–39.71
				12%	1,227	784–1,606	0.64	0.41–0.84	160	103–210	5.67	3.63–7.41
				80%	8,143	5,213–10,636	4.28	2.74–5.59	1,059	681–1,379	37.43	24.07–48.73
Combined	62–85%	30–75%	80%^d^	65%	10,441	8,014–12,514	5.48	4.21–6.56	1,355	1,044–1,624	47.91	36.93–57.29
			70%^d^	12%	9,650	8,393–10,717	5.07	4.41–5.63	1,253	1,089–1,397	44.31	38.59–49.14
			95%^d^	80%	10,733	7,782–13,219	5.64	4.09–6.93	1,393	1,010–1,714	49.24	35.76–60.53

CI: credible intervals; mAbs: monoclonal antibody nirsevimab; n/a: not applicable; RSV: respiratory syncytial virus.

^a^ Lombardy population size: 9,965,046 inhabitants as at 1 January 2022 [22].

^b^ ‘mAb’ refers to nirsevimab administration to infants; ‘vaccination’ refers to RSVpreF vaccine administration to pregnant women.

^c^ Efficacy estimates for the monoclonal antibody nirsevimab [18] and for the RSVpreF vaccine [13].

^d^ These percentages are applied to the children whose mothers did not receive the vaccine during pregnancy.

In the main analysis, we assumed that the protection provided by mAbs lasts only for the first season after administration. Such assumption is in line with the available evidence suggesting that there is no residual protection in subsequent seasons [11,24]. However, to explore the potential effect of considering different plausible assumptions on the long-term immunity provided by mAb administration, we considered two alternative hypotheses. In a first sensitivity analysis (SA1), we assume that infants who received mAbs develop the same persistent partial immunity as the individuals who had experienced a primary RSV infection – specifically, a 23% lower risk of getting infected in the subsequent seasons. This assumption is based on the findings of Wilkins et al. [10], which suggested that mAbs allow for the development of an immune response to RSV. In a second sensitivity analysis (SA2), in addition to the 23% reduced susceptibility considered in the sensitivity analysis SA1, we also assume a persisting long-term protection against hospitalisation for RSV infections occurring among individuals who have received mAbs in any preceding season. This long-term protection is assumed to be equal to the efficacy of mAbs against RSV infection, as clinical trials [18] report no significant differences in efficacy estimates between reductions in medically attended RSV-associated infections and hospitalisations.

Finally, we consider different approaches to model the effect of mAbs and vaccine in reducing the risk of infection in immunised infants. In the main analysis, we consider an ‘all-or-nothing’ approach which assumes that the immunising intervention provides complete protection to a fraction of the individuals who received the treatment and no protection to the remaining part of the treated population. In this case, the proportion of individuals receiving full protection by the intervention is thus computed as the coverage times the efficacy of the intervention. For sensitivity analysis, we assume that all immunised individuals have a reduced risk of infection on account of mAbs and maternal vaccine administration (‘leaky’ approach; sensitivity analyses L1 and L2). Under the leaky assumption, the probability of primary infection among individuals aged less than 1 year (*a* = 0) in season y becomes:


$$P_{0,y}^{1}\left(\;r_{int}\right)=\left(1-e^{-\left(1-\;r_{int}\;\right)∙\lambda \left(0,y\right)}\right)\;(5)$$

where *r_int_* is the reduction in the risk of infection provided by either mAbs or vaccine (*r_int_* = 0 for non-immunised individuals). The value of *r_int_* was chosen to reconstruct the efficacy estimates of nirsevimab and RSVpreF vaccine from the clinical trials [13,18]. Specifically, *r_mAbs_* was computed to match *ε_mAbs_* = 1 − *RR* [18], where *RR* is the RR of observing a RSV medically-attended case among immunised individuals compared with non-immunised individuals and *r_vax_* was computed to match *ε_vax_* = 1 – *P/(1 – P)* [13], where *P* is the ratio of vaccinated cases to the total number of cases observed in a randomised clinical trial with a 1:1 ratio (sensitivity L1). We also conducted a sensitivity analysis considering ‘leaky’ interventions, under the simpler assumption that *r_mAbs_* = *ε_mAbs_* and *r_vax_* = *ε_vax_* (sensitivity L2). More mathematical details are provided in the Supplementary section ‘Modelling the effect of mAbs and maternal vaccine’.

## Results

Model estimates show that after the strong fluctuations in RSV circulation driven by the COVID-19 pandemic [15] (2020–22), the RSV susceptibility profile and the age-specific RSV incidence in Lombardy have reverted to levels comparable to those observed in the pre-pandemic period (season 2018/19).

**Figure 1:**
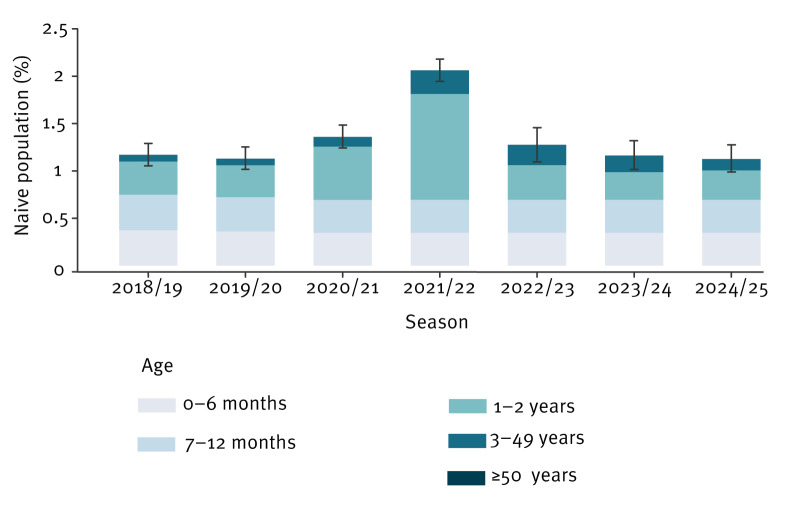
Model estimates of RSV susceptibility over time under the baseline scenario (no interventions), Lombardy, Italy, 2018/19–2024/25 seasons

Our estimates suggest that the proportion of naive individuals, i.e. individuals who have never experienced RSV infection, changed from 1.17% (95% CI: 1.06–1.29) before the 2018/19 season to 2.06% (95% CI: 1.95–2.18) before the 2021/22 season [15] and returned to 1.12% (95% CI: 1.00–1.27) just before the 2024/25 season (Figure 1).

RSV: respiratory syncytial virus.

Estimated percentage of the population naive to RSV infection at the start of each season. Bars represent mean estimates, and the lines represent 95% credible interval.

**Figure 2:**
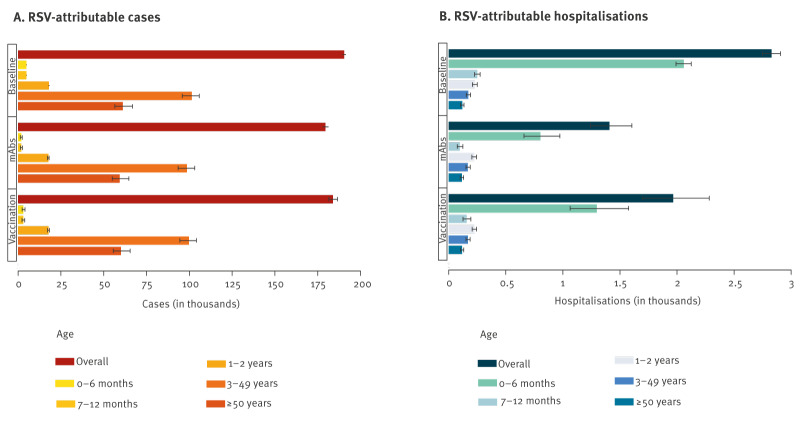
Impact of different immunisation strategies on the age-stratified incidence of RSV-attributable cases and hospitalisations, Lombardy, Italy, 2024/25 season

In the absence of interventions (representing the baseline scenario), at the end of the 2024/25 season, the estimated number of medically attended RSV cases stands at 190,470 (95% CI: 189,600–191,309), with 9,800 cases (95% CI: 9,704–9,896) occurring among infants, representing on average 5.15% of the total cases (Figure 2A). The incidence of RSV hospitalisation in 2024/25 is estimated at 2,828 (95% CI: 2,752–2,906) hospitalised individuals, with 2,309 hospitalisations (95% CI: 2,238–2,378) occurring among infants (on average, 81.7% of the total hospitalisations) (Figure 2B). Under the assumption of a stable RSV transmission rate in the subsequent seasons and in the absence of immunising interventions, both the age-specific incidence of RSV-associated LRTI cases and hospitalisations are expected to remain stable in the future (Figure 1). This stability is also reflected in the uncertainty surrounding model projections of cases and hospitalisations in the future. Model estimates of RSV-attributable cases and hospitalisations from 2022 to 2025 are provided in Supplementary Figure S1.

RSV: respiratory syncytial virus.

Model estimates of the age-specific number of (A) RSV-attributable cases and (B) RSV-attributable hospitalisations for the 2024/25 season when assuming no interventions (‘baseline’ scenario), mAb administration to 80% of infants (‘mAbs’), and vaccination of 65% of pregnant women (‘vaccination’).

### Main analysis

The simulation results of the introduction of routine prophylaxis in the 2024/25 season, either based on mAbs or maternal immunisation, suggest that both products have the potential to substantially reduce the overall burden caused by severe RSV infections (see Figure 2). Compared with the baseline scenario, mAb administration to 80% of infants is estimated to reduce the overall number of reported RSV-attributable cases by 5.75% (95% CI: 4.97–6.40) and the overall number of RSV-associated hospitalisations by 50.2% (95% CI: 43.5–55.8). Accordingly, this would result in 179,520 (95% CI: 178,010–181,157) cases overall and 1,408 (95% CI: 1,246–1,602) hospitalisations per season (see Figure 2 and the Table). Among infants (representing the target age group), routine mAb administration is expected to reduce the number of hospitalisations caused by RSV from 2,309 (95% CI: 2,238–2,378) to 902 (95% CI: 744–1,090) hospitalised individuals, therefore averting 61.0% of RSV hospitalisations in this age group on average. The numbers and the percentages of averted cases and hospitalisations among infants are reported in Supplementary Table S2.

Compared with the baseline scenario, vaccine administration to 65% of pregnant women is estimated to reduce the number of RSV-attributable cases in the overall population by 3.48% (95% CI: 2.23–4.54), resulting in 183,846 (95% CI: 181,677–186,309) cases per season (see Figure 2A and the Table), and the number of RSV-associated hospitalisations by 30.5% (95% CI: 19.6–39.7), resulting in 1,966 (95% CI: 1,705–2,281) hospitalisations per season (see Figure 2B and the Table). Maternal vaccination is estimated to reduce the number of hospitalisations in infants from 2,309 (95% CI: 2,238–2,378) to 1,455 (95% CI: 1,195–1,765) hospitalised individuals per season (37.7% of hospitalisations averted on average; see Supplementary Table S2). Both mAb administration to infants and maternal vaccination result in only a marginal reduction in RSV-attributable cases in age groups older than 1 year, because of the relatively small contribution of infants to RSV transmission and the consequently limited indirect effect on the infection circulation (see Figure 2 and Supplementary Table S2).

### Alternative scenarios and sensitivity analyses

Exploring coverage values ranging from 70% to 95% for mAbs, the average proportion of RSV hospitalisations averted in the general population ranges from 44.0% (95% CI: 38.2–48.9) to 59.5% (95% CI: 51.6–66.0). For maternal vaccination with coverage ranging from 12% to 80%, the percentage of RSV hospitalisations averted is expected to range from 5.67% (95% CI: 3.63–7.41) to 37.4% (95% CI: 24.1–48.7).

**Figure 3:**
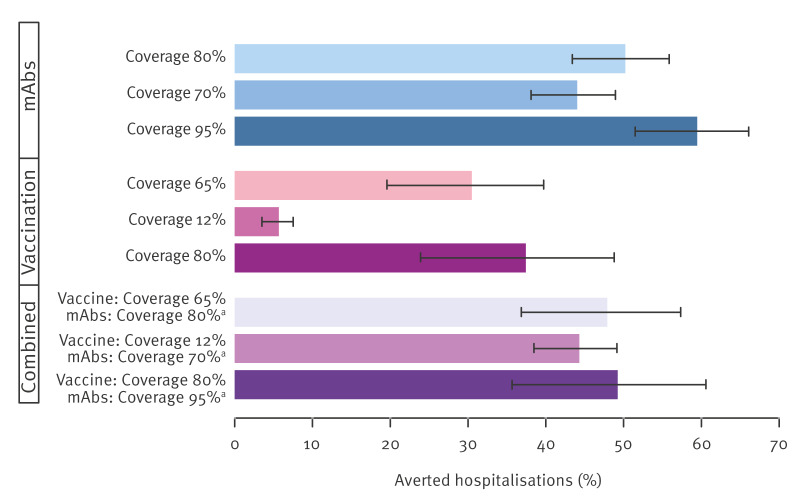
Expected proportion of RSV hospitalisations averted in the overall population by different immunisation strategies for varying coverage levels, Lombardy, Italy, 2024/25 season

The estimated average percentage of RSV hospitalisations averted by combining maternal vaccination and the administration of mAbs only in infants of unvaccinated mothers ranges between 44.3–49.2% in the general population (53.8–59.8% in infants), depending on coverage levels assumed in the simulations (see Figure 3 and the Table). The lower average effectiveness of this strategy, compared with mAb administration alone to more than 80% of infants, is due to the lower efficacy we assumed for the RSVpreF vaccine compared with nirsevimab (30–75% vs 62–85%) and the fact that in the combined scenario nirsevimab is given only to infants born to unvaccinated mothers. The Table and Supplementary Table S2 summarise the expected impact of mAbs and maternal vaccination according to the explored scenarios.

mAbs: monoclonal antibodies; RSV: respiratory syncytial virus.

Bars display the percentage of averted RSV hospitalisations by nirsevimab administration to infants (‘mAbs’), RSVpreF vaccine administration to pregnant women (‘vaccination’), and by a strategy combining vaccination and mAb administration (‘combined’), with respect to the no intervention (‘baseline’) scenario. Bars represent the mean estimates; lines represent the 95% credible interval.

^a^ These percentages are applied to the children whose mothers did not receive the vaccine during pregnancy.

Projections of model estimates towards a longer time horizon (2029/30) suggest that the number of hospitalisations averted by seasonal administration of mAbs in infancy and/or by maternal vaccination would remain constant in the future. However, assuming a life-long partial protection for individuals who received mAbs in the preceding seasons, a further reduction of RSV hospital burden is expected within the next 5 years. Specifically, by assuming that mAb administration results in a 23% lower risk of infection in the following seasons (see sensitivity analysis SA1), an additional 1.40% average reduction of hospitalisations (50.4% averted hospitalisations compared with the baseline scenario) in the general population is estimated by the 2029/30 season (see Supplementary Figure S5 for estimates of hospitalisations following routine mAb administration under sensitivity analyses SA1 and SA2). This further reduction increases on average to 10.9% (55.1% averted hospitalisations compared with the baseline scenario) when also considering a lower risk of hospitalisation upon infection for individuals who received mAbs in the past (sensitivity analysis SA2; Supplementary Figure S5). Alternative assumptions on the mode of action of mAbs and maternal vaccination yield consistent impact estimates with those obtained under our main assumptions (see Supplementary Figures S6–S7 for estimates of averted hospitalisations under sensitivity analyses L1 and L2).

## Discussion

Prophylactic campaigns based on mAb administration in infancy or maternal vaccination have the potential to markedly reduce the hospital burden associated with RSV. In Italy, non-pharmaceutical interventions implemented during the COVID-19 pandemic almost completely suppressed RSV circulation during the 2020/21 season, changing the immunity profile of the population and resulting in a rise of RSV-related hospitalisations in the following season [15]. In this study, we quantified the number of cases and hospitalisations that could be averted in Lombardy by the seasonal administration of nirsevimab to infants and the regular vaccination of pregnant women with RSVpreF vaccine. While several studies have investigated the effects of different immunisation strategies based on mAbs [26-36] and maternal vaccination [31-37], to the best of our knowledge, this is the first data-driven analysis to evaluate the potential impact of nirsevimab [18] and RSVpreF vaccine [13] in Italy, accounting also for changes in the age-specific immunity profile occurred during the COVID-19 pandemic. The analysis of alternative coverage levels as well as of strategies combining maternal vaccination with mAb administration provides a comprehensive picture of the plausible impact of routine prophylaxis of infants on the disease burden of RSV in Italy.

Despite a marginal effect on the overall RSV circulation, we estimated that the administration of mAbs to 80% of infants is expected to avert on average around 60% of hospitalisations in infants and 50% in the overall population. In line with available evidence [11,24], our main analysis assumes that mAb protection lasts only for the first season after administration. However, the sensitivity analyses conducted showed that the impact of seasonal mAb administration would remain relatively constant in the near future, regardless of potential uncertainty about the residual protection provided by mAbs in the seasons following their administration. Our findings are line with results from previous modelling studies [26,31,33], including a recent analysis conducted in Canada [35] that suggests an average 63% reduction in paediatric ward and intensive care unit admissions among infants following nirsevimab administration at 80% coverage level. Additionally, a study conducted in Luxembourg after the start of the nirsevimab campaign at the beginning of season 2023/24 [38] found that neonatal coverage of 84% resulted in an average 69% reduction in RSV-associated hospitalisations among infants < 6 months old compared to the same period in the previous season, consistently with our model estimates. 

We estimated that maternal vaccination with RSVpreF vaccine at 65% coverage would on average reduce the RSV hospitalisations by 38% among infants and by 31% in the overall populations; this is in good agreement with the 38% reduction in infant hospital admissions estimated for Spain when considering the administration of RSVpreF vaccine to 70% of pregnant women [37]. Consistent with other studies comparing nirsevimab and RSVpreF vaccine impact [31,32,35], our findings suggest that the administration of mAbs is more effective in reducing the number of hospitalisations caused by RSV infection. This result is strongly driven by the higher efficacy and higher uptake assumed for mAbs compared with the maternal vaccine. Monoclonal antibodies are estimated to be more effective than the maternal vaccine even when assuming the same coverage (50.7% vs 38.2% averted hospitalisations in the general population on average), because of the higher levels of protection reported in the clinical trials for nirsevimab compared with the RSVpreF vaccine [12,13,18,19]. However, uptake levels associated with the two immunising strategies are expected to be markedly different. This expectation arises from the high coverage of mAbs recently achieved in Spain [20], the unprecedented demand for nirsevimab recorded in the United States [39], a generally higher vaccine hesitancy reported among pregnant women [40], and the relatively low uptake levels observed during pregnancy for other vaccines routinely administered in Lombardy. Our findings show that, even considering the lowest estimate for mAb efficacy, seasonal routine nirsevimab prophylaxis based on uptake levels similar to early observations in Spain during 2023 among non-high-risk individuals can lead to more than 40% averted hospitalisations.

Our analysis has limitations. Firstly, the efficacy against medically attended LRTI reported in the clinical trials for nirsevimab [18] and RSVpreF vaccine [13] were assumed as proxies for their efficacy against RSV infection. This assumption could have led to an overestimation of the efficacy against RSV and hence to larger than expected population-level impacts for both mAbs and maternal vaccination. Secondly, we also assumed that the timing of administration was consistent with the one adopted during the clinical trials, and that protection lasts for the entire first RSV season for both mAbs and the maternal vaccine. Protection from mAbs has been assessed through 150 days after the injection [18,19], meaning protection throughout the entire first RSV season could be achieved with an optimal administration timing [41]. Evidence on the duration of protection from maternal vaccination is sparse [41]. Thirdly, from a modelling perspective, the catalytic approach used in this study does not allow us to consider changes in the transmission dynamics and assumes homogeneous mixing in the population. This means that: (i) the progressive waning of protection provided by mAbs, maternal vaccination and passive transfer of natural maternal immunity are only implicitly considered; and (ii) indirect effects on specific segments of the population, e.g. in the older people (≥ 60 years old), might have been underestimated [42]. According to our estimates, both mAbs and maternal vaccination are expected to result in a negligible reduction in RSV cases and hospitalisations among other at-risk age groups, including the older people. Although we found a relatively low impact of prophylactic strategies targeting infants on the overall circulation of RSV, indirect benefits from an overall lower pressure on the healthcare system are expected. Fourthly, the reduction in susceptibility following primary RSV infection was assumed to be constant over time, neglecting its potential waning. However, since most hospitalisations occur among naive infant individuals, and we found that prophylactic measures would produce only a marginal reduction of incidence in older individuals, we expect that similar results would be obtained even if waning protection from a previous infection were considered. Fifthly, higher costs are associated with the administration of nirsevimab (ca 230 EUR per dose [43]) compared with the RSVpreF vaccine (ca 180 EUR per dose [44]), yet the proposed analysis does not include an evaluation of the overall costs associated with the two potential prophylactic strategies. Finally, we assumed that contact patterns and RSV incidence for seasons beyond 2024 would be restored to pre-pandemic levels and that the age distribution and population size will not dramatically change in the next 5 years. Possible changes in the RSV circulation should be therefore monitored in the near future. Despite these limitations, our study provides estimates of the impact of introducing seasonal immunoprophylactic campaigns for RSV based on newly approved mAbs and maternal vaccine, while considering changes in the RSV epidemiology because of the COVID-19 pandemic.

## Conclusions

Our results suggest that nirsevimab administration in infancy could play a major role in reducing the burden of RSV, while maternal vaccination could be implemented as a complementary measure to achieve higher protection levels in infants or indicated in mothers of infants with risk factors for adverse outcomes from RSV infection. These findings, in addition to cost-effectiveness evaluations, could support public health policymakers in the decision to include nirsevimab or the fRSVpreF vaccine into childhood immunisation programmes.

## Supplementary Data

522000 Supplement
