# Supplementary material for: Impact of routine prophylaxis with monoclonal antibodies and maternal immunisation to prevent respiratory syncytial virus hospitalisations, Lombardy region, Italy, 2024/25 season
Source: Euro Surveill. 2025 Apr 10;30(14):2400637. doi: 10.2807/1560-7917.ES.2025.30.14.2400637 (PMC11987492; doi:10.2807/1560-7917.ES.2025.30.14.2400637)
Supplement: Supplement [file 24-00637_POLETTI_Supplement.pdf]

This supplementary material is hosted by Eurosurveillance as supporting information alongside the article "Impact of routine prophylaxis with monoclonal antibodies and maternal immunisation to prevent respiratory syncytial virus hospitalisations, Lombardy region, Italy, 2024/25 season", on behalf of the authors, who remain responsible for the accuracy and appropriateness of the content. The same standards for ethics, copyright, attributions and permissions as for the article apply. Supplements are not edited by Eurosurveillance and the journal is not responsible for the maintenance of any links or email addresses provided therein.

# Supplementary Material

**Table of contents**

Methodological details and data .......... 2
- Mathematical model .......... 2
- Data description and model fit .......... 4
- Sensitivity analyses .......... 7
  - MAbs effect in the long term .......... 7
  - Modelling the effect of mAbs and maternal vaccine .......... 8

Additional results and analyses .......... 11
References .......... 14

# Methodological details and data

## Mathematical model

The risk of RSV infection is simulated in a population stratified by age (namely, 100 age groups from 0 to 99 years, plus one age group for individuals aged 100 years or older). The force of infection (FOI) $\lambda(a,y)$ is assumed to vary by age $(a)$ and season $(y)$ and to be piecewise constant for three age-groups (0-4, 5-14, and 15+ years), as proposed by Ang et al. [1]:

$$\lambda(a,y) = \begin{cases} \lambda_y^{0-4} \;, & a \le 4 \\ \lambda_y^{5-14}, & 4 < a \le 14 \\ \lambda_y^{15+} \;, & a > 14 \end{cases} \quad (1)$$

In the absence of immunising interventions, we assume that the FOI for an individual of age $a$ in season $y$ is directly proportional to the contact rate of individuals of age $a$ in season $y$ $(\beta(a,y))$ and to the overall number of RSV cases ascertained in season $y$ $(i(y))$, normalised such that $\max_y i(y) = 1$:

$$\lambda(a,y) = \beta(a,y) \cdot i(y) \quad (2)$$

The transmission rate in the first age group implicitly accounts for temporary protection against RSV infection in infants provided by passive transfer of maternal immunity.
We assume that the administration of monoclonal antibodies (mAbs) and maternal vaccine provides an indirect reduction on the FOI, driven by the estimated contribution of infants to the to the overall RSV incidence. The FOI is then described as:

$$\begin{aligned} &\lambda(a,y) = \beta(a,y) \cdot i(y) \cdot [x(0,y-1) \cdot (c_{mAbs}(y)(1-\varepsilon_{mAbs}) \\ &+c_{vax}(y)(1-\varepsilon_{vax})) \qquad (3) \\ &+1 - x(0,y-1)(c_{mAbs}(y) + c_{vax}(y))] \end{aligned}$$

where $c_{mAbs}(y)$ and $c_{vax}(y)$ are the coverage levels of mAbs and vaccine in season $y$, $\varepsilon_{mAbs}$ and $\varepsilon_{vax}$ are the efficacy levels against RSV infection of mAbs and vaccines respectively, and $x(0,y-1)$ is the proportion of RSV cases that occurred in individuals aged 0-1 years in the counterfactual scenario (i.e. when no interventions are implemented). The FOI reduces to $\lambda(a,y) = \beta(a,y) \cdot i(y)$ when considering counterfactual simulations and for seasons preceding the introduction of mAbs administration or vaccination. We assumed that the seasons after 2021-22 are characterised by the FOI estimated for the pre-COVID-19 season 2018–2019.
From equations 1-3 of the main text, the model estimates the number of all infections, reported and non-reported, occurring at any age of interest. This quantity was matched for the years 2018-2022 to observed data on medically attended cases of LTRI and hospitalisations caused by RSV as detailed in the following sections.

***Expected number of RSV-attributable cases and hospitalisations***

From equation (4) in the main text, we can derive the joint probability of getting infected by RSV in season $y$ and receiving mAbs in the first year of life:

$$P_{a,y}(\varepsilon_{mAbs}) = c_{mAbs}(y-a)\left(P^1_{a,y}(\varepsilon_{mAbs}) + P^2_{a,y}(\varepsilon_{mAbs})\right) \quad (4)$$

Similarly, the joint probability of getting infected by RSV in season $y$ and receiving protection in the first year of life from maternal vaccination, and the joint probability of getting infected by RSV in season $y$ and not receiving any immunisation are defined as:

$$P_{a,y}(\varepsilon_{vax}) = c_{vax}(y-a)\left(P^1_{a,y}(\varepsilon_{vax}) + P^2_{a,y}(\varepsilon_{vax})\right) \quad (5)$$

$$P_{a,y}(0) = (1 - c_{mAbs}(y-a) - c_{vax}(y-a))\left(P^1_{a,y}(0) + P^2_{a,y}(0)\right) \quad (6)$$

We define the average probability of getting infected and reported to the surveillance system in season $y$ for an individual in age-group $g$ who did not receive any treatment, as

$$\pi^{y,Surv}_{g,n} = \rho^{Surv}_g \frac{\sum_{a\in g} P_{a,y}(0) N^y_a}{\sum_{a\in g} N^y_a} \quad (7)$$

where $\rho^{Surv}_g$ represents the joint probability that an RSV infection in age-group $g$ develops symptoms and is reported to surveillance system after infection, and $N^y_a$ is the number of individuals of age $a$ in season $y$. Analogously, the average probability to get infected and reported to surveillance system in season $y$ for an individual in age-group $g$ who received mAbs in the past and received protection from maternal vaccination are respectively:

$$\pi^{y,Surv}_{g,mAbs} = \rho^{Surv}_g \frac{\sum_{a\in g} P_{a,y}(\varepsilon_{mAbs}) N^y_a}{\sum_{a\in g} N^y_a} \quad ; \quad \pi^{y,Surv}_{g,vax} = \rho^{Surv}_g \frac{\sum_{a\in g} P_{a,y}(\varepsilon_{vax}) N^y_a}{\sum_{a\in g} N^y_a} \quad (8)$$

The expected number of ascertained RSV cases in age-group $g$ during season $y$ among individuals who received mAbs in infancy, who have been protected from maternal vaccination, and who did not receive any protection are therefore:

$$\Lambda^{Surv,mAbs}_{g,y} = \pi^{y,Surv}_{g,mAbs} N^y_g ; \quad \Lambda^{Surv,vax}_{g,y} = \pi^{y,Surv}_{g,vax} N^y_g ; \quad \Lambda^{Surv,n}_{g,y} = \pi^{y,Surv}_{g,n} N^y_g \quad (9)$$

We assume that the probability of being reported remains constant in time but differs across the age-groups. Age-groups considered are: 0-4 years, 5-14 years, 15-64 years, and 65+ years.

Similarly, we define the average probability of an individual in age-group $g$ who never received mAbs or protection from maternal vaccination to get infected and hospitalised in season $y$ as

$$\pi^{y,Hosp}_{g,n} = \rho^{Hosp}_g \frac{\sum_{a\in g} P^y_a(0) N^y_a}{\sum_{a\in g} N^y_a} \quad (10)$$

where $\rho_g^{Hosp}$ is the probability of hospitalisation due to RSV in age-group $g$ for individual who never received mAbs.

The average probability to get infected and hospitalised in season $y$ for an individual in age-group $g$ who received mAbs in the past and received protection from maternal vaccination is respectively:

$$\pi_{g,mAbs}^{y,Hosp} = \rho_g^{Hosp} \frac{\sum_{a \in g} P_a^y(\varepsilon_{mAbs}) N_a^y}{\sum_{a \in g} N_a^y} \quad ; \quad \pi_{g,vax}^{y,Hosp} = \rho_h^{Hosp} \frac{\sum_{a \in g} P_a^y(\varepsilon_{vax}) N_a^y}{\sum_{a \in g} N_a^y} \qquad (11)$$

The expected number of RSV hospitalisations in age-group $g$ during season $y$ among individuals who received mAbs in infancy, who has been protected from maternal vaccination, and who did not receive any protection is defined as

$$\Lambda_{g,y}^{Hosp,mAbs} = \pi_{g,mAbs}^{y,Hosp} N_g^y; \quad \Lambda_{g,y}^{Hosp,vax} = \pi_{g,vax}^{y,Hosp} N_g^y; \quad \Lambda_{g,y}^{Hosp,n} = \pi_{g,n}^{y,Hosp} N_g^y \qquad (12)$$

We assume that the probability of hospitalisation for individual who never received mAbs remains constant in time but differs across the following age-groups: 0-6 months, 7-12 months, 1-2 years, 2-3 years, and 3+ years.

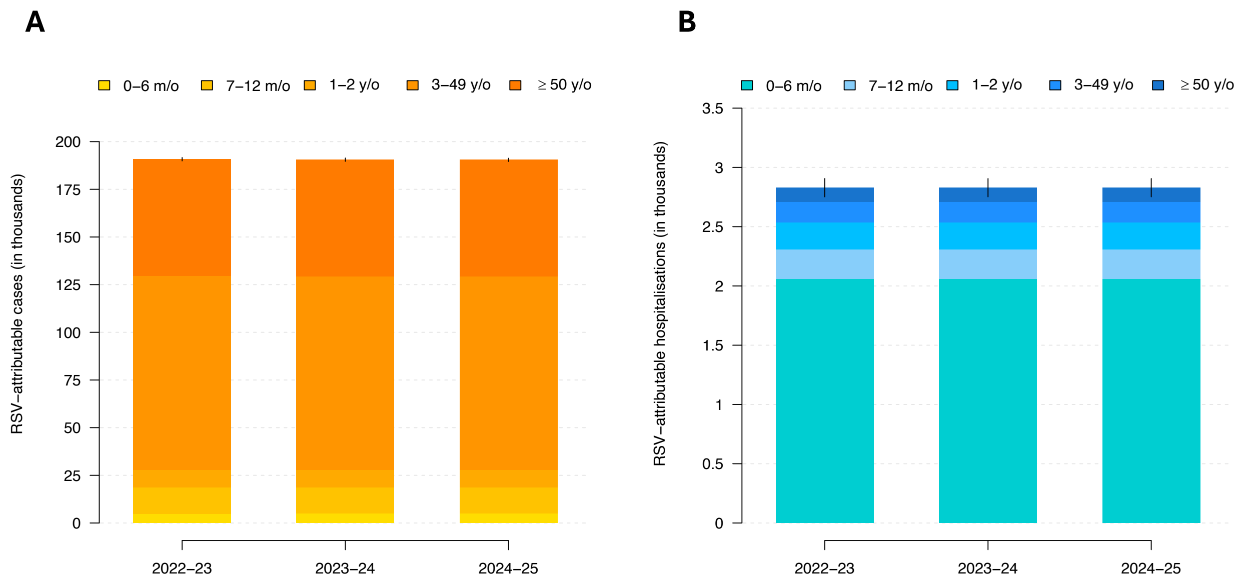


**Figure S1. Model estimates of RSV-attributable cases and hospitalisations from 2022 to 2025 in Lombardy, Italy, under the baseline scenario (no interventions).** **A)** Age-specific estimates of the number of RSV-attributable cases per season in the region. **B)** Age-specific estimates of the number of hospitalisations caused by RSV circulation per season in the region. Bars: mean estimates; lines: 95% CI.

## Data description and model fit

The model is calibrated on age-specific records of RSV-attributable cases and hospitalisations ascertained in the Lombardy region between 2018 and 2022.

The number of medically attended RSV cases per age-group from 2018 to 2022 was derived from age-specific aggregated time-series of syndromic and virological surveillance data extracted from the Italian influenza surveillance network (RespiVirNet, formerly InfluNet) [2]. RespiVirNet is a sentinel network of general practitioners (GPs) and paediatricians that reports weekly numbers of outpatients seeking care for influenza-like illness (ILI) along with the number of cases tested for respiratory viruses including RSV. The ILI and virological data were collected for four age groups: 0–4, 5–14, 15–64, and 65+ years. RespiVirNet data were used to estimate the number of RSV attributable cases per age-group and season, by multiplying the age-specific ILI incidence, the seasonal RSV test positivity rate, and the size of the Lombardy population [3]. The age-specific number of hospitalisations caused by RSV infections for each season from 2018 to 2022 were provided by the official authorities of Lombardy region [4]. The number of weekly hospital discharges was made available for the following age -groups: 0–6 months, 7–12 months, yearly age-groups up to age 80, and an aggregated group for individuals 81+ years. Further details on the data are reported in [1]. Figures S2 and S3 show the model fit to the number and age-distribution of RSV-attributable cases and hospitalisations due to RSV. Figure S4 shows model estimates for the absolute number of cases and hospitalisations cumulatively observed between 2018 and 2022. Although non-negligible differences between the estimated and observed absolute number of hospitalisations in infants aged 0–6 months were identified for the 2018–19 season, the model accurately captures both the distribution of hospitalisations across different age groups (see Figure S3) and the cumulative age-specific burden of hospitalisations from 2018 to 2022 (see Figure S4). Therefore, we do not expect these differences to substantially impact the analysis results, either in terms of the ranking of the best prophylaxis strategies (with mAbs being more effective than the RSVpreF vaccine) or the percentage of averted hospitalisations.

The posterior distributions of the model parameters used in all the analyses presented in the main text and in the Supplementary Material are summarized in Table S1.

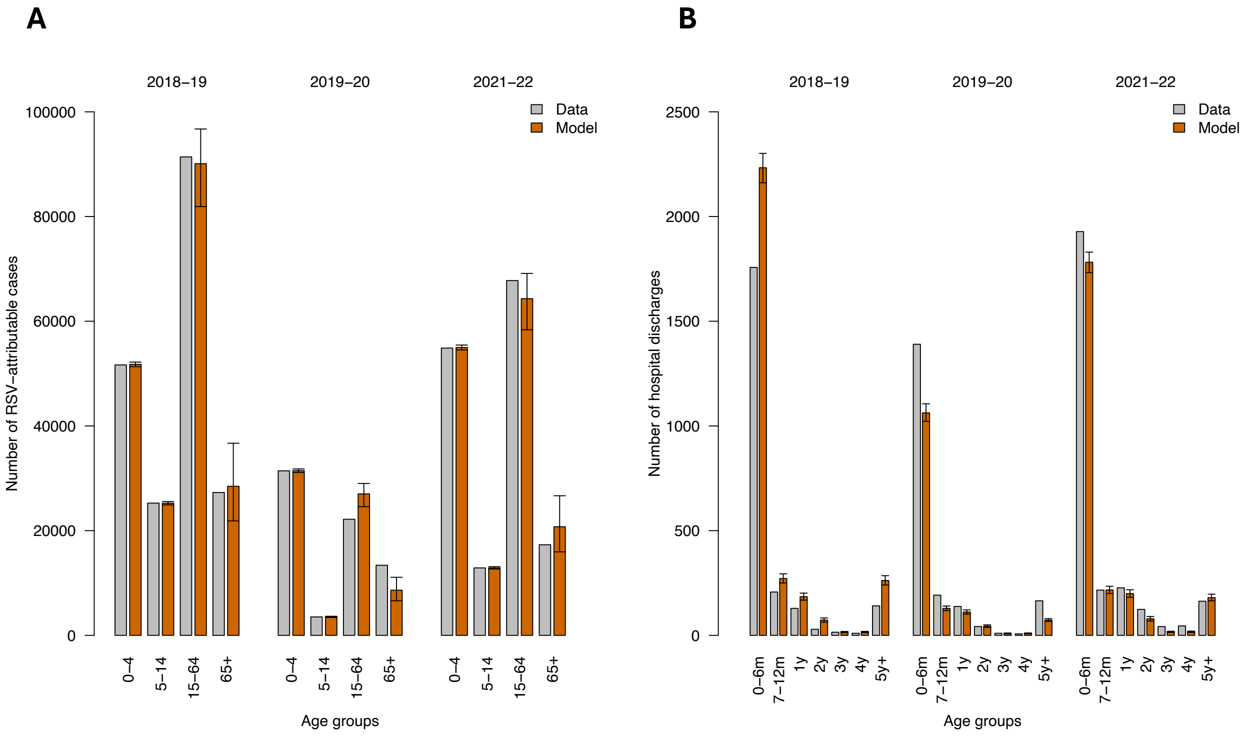

**Figure S2.** Observed and modelled age-specific number of **A)** RSV-attributable cases ascertained in the Lombardy Region over time and **B)** hospital discharges associated with RSV recorded by the Welfare General Directorate of the Lombardy Region across the corresponding seasons. Bars: data (grey) or mean estimate (brown), whiskers: 95% CI.

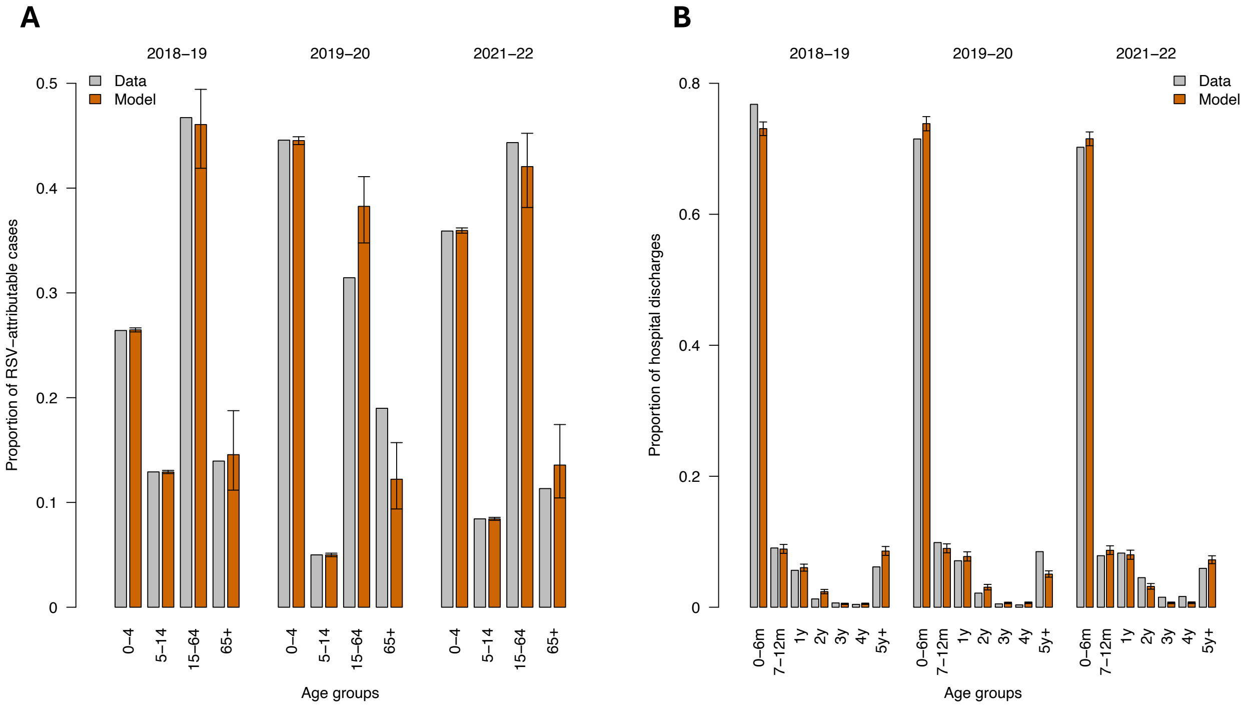


**Figure S3.** Observed and modelled age-distribution of **A)** RSV-attributable cases ascertained in the Lombardy Region over time and **B)** hospital discharges associated with RSV recorded by the Welfare General Directorate of the Lombardy Region across the corresponding seasons. Bars: data (grey) or mean estimate (brown), whiskers: 95% CI.

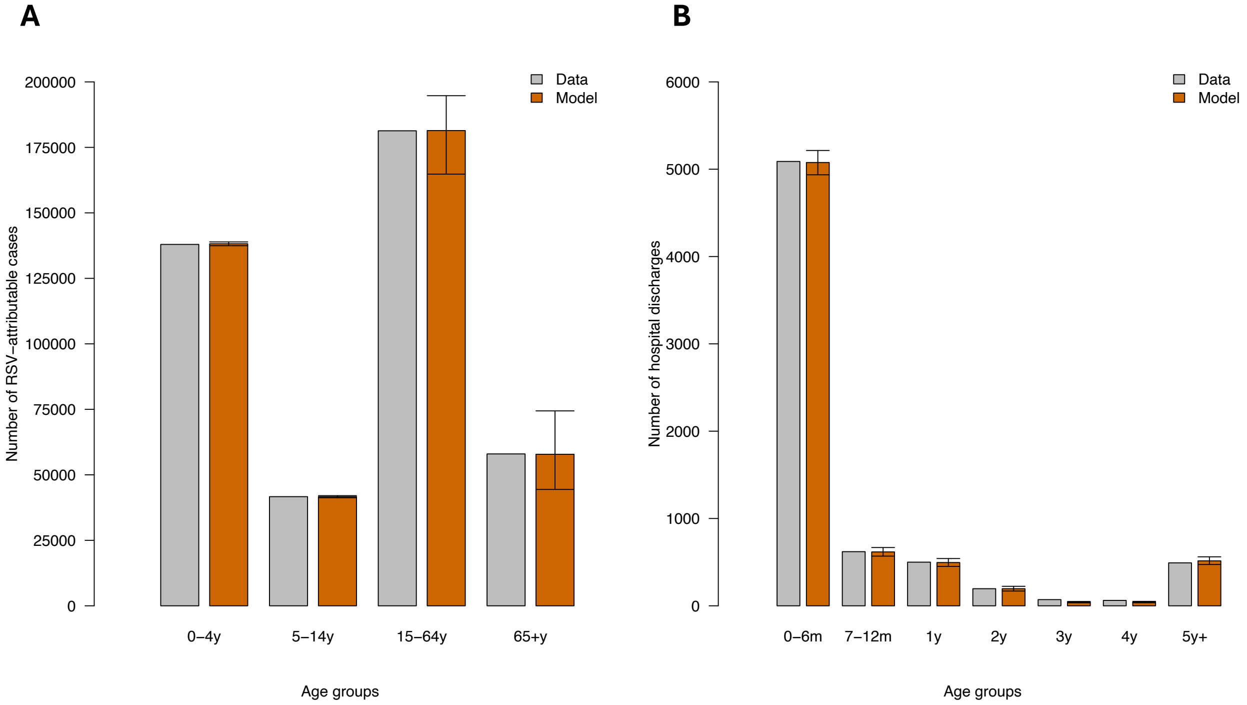


**Figure S4.** Observed and modelled age-specific number of **A)** RSV-attributable cases and

**B)** hospitalisations cumulatively observed between 2018 and 2022 in the Lombardy Region. Bars: data (grey) or mean estimate (brown), whiskers: 95% CI.

**Table S1.** Posterior distributions of model parameters (average and 95% credible intervals).

| Parameter | Posterior distribution | Description |
|---|---|---|
| $\beta_{2018-19}^{0-4}$ | 1.12 [0.97 - 1.30] | Transmission rate for individuals aged 0-4 years in season 2018-2019 |
| $\beta_{2018-19}^{5-14}$ | 0.17 [0.14 - 0.24] | Transmission rate for individuals aged 5-14 years in season 2018-2019 |
| $\beta_{2018-19}^{15+}$ | 0.11 [9.2 x $10^{-2}$ - 0.14] | Transmission rate for individuals aged 15+ years in season 2018-2019 |
| $\beta_{2019-20}^{0-4}$ | 1.58 [1.42 - 1.78] | Transmission rate for individuals aged 0-4 years in season 2019-2020 |
| $\beta_{2019-20}^{5-14}$ | 6.3 x $10^{-2}$ [5.0 x $10^{-2}$ - 8.6 x $10^{-2}$] | Transmission rate for individuals aged 5-14 years in season 2019-2020 |
| $\beta_{2019-20}^{15+}$ | 8.9 x $10^{-2}$ [7.5 x $10^{-2}$ - 0.11] | Transmission rate for individuals aged 15+ years in season 2019-2020 |
| $\beta_{2021-22}^{0-4}$ | 1.76 [1.51 - 2.11] | Transmission rate for individuals aged 0-4 years in season 2021-2022 |
| $\beta_{2021-22}^{5-14}$ | 0.11 [8.8 x $10^{-2}$ - 0.15] | Transmission rate for individuals aged 5-14 years in season 2021-2022 |
| $\beta_{2021-22}^{15+}$ | 0.10 [8.4 x $10^{-2}$ - 0.12] | Transmission rate for individuals aged 15+ years in season 2021-2022 |
| $\rho_{0-4}^{Surv}$ | 0.21 [0.19 - 0.23] | Ascertainment rate for individuals aged 0-4 years |
| $\rho_{5-14}^{Surv}$ | 0.22 [0.16 - 0.27] | Ascertainment rate for individuals aged 5-14 years |
| $\rho_{15-64}^{Surv}$ | 0.18 [0.14 - 0.21] | Ascertainment rate for individuals aged 15-64 years |
| $\rho_{65+}^{Surv}$ | 0.16 [0.12 - 0.19] | Ascertainment rate for individuals aged 65+ years |
| $\rho_{0-6m}^{Hosp}$ | 8.9 x $10^{-2}$ [8.0 x $10^{-2}$ - 9.7 x $10^{-2}$] | Hospitalisation rate for individuals aged 0-6 months |
| $\rho_{7-12m}^{Hosp}$ | 1.1 x $10^{-2}$ [9.5 x $10^{-3}$ - 1.2 x $10^{-2}$] | Hospitalisation rate for individuals aged 7-12 months |
| $\rho_{1}^{Hosp}$ | 3.9 x $10^{-3}$ [3.4 x $10^{-3}$ - 4.4 x $10^{-3}$] | Hospitalisation rate for individuals aged 1 year |
| $\rho_{2}^{Hosp}$ | 1.5 x $10^{-3}$ [1.3 x $10^{-3}$ – 1.8 x $10^{-3}$] | Hospitalisation rate for individuals aged 2 years |
| $\rho_{3+}^{Hosp}$ | 3.2 x $10^{-4}$ [2.7 x $10^{-4}$ - 3.8 x $10^{-4}$] | Hospitalisation rate for individuals aged 3+ years |

*Transmission rates for season 2020-2021 were not calibrated as the incidence in season 2020-2021 was 0.

# Sensitivity analyses

## MAbs effect in the long term

We conducted two sensitivity analyses, considering only the administration of monoclonal antibodies (i.e., $c_{vax}(y) = 0$).

In the first sensitivity analysis (SA1) we assume that the administration of mAbs also provides a partial reduction in the risk of infection in the subsequent seasons following the administration. Specifically, we assume that all individuals of age $a \geq 1$ who have received mAbs in the previous years are exposed to the risk of infection as those who have already experienced a primary RSV infection (23% lower risk of infection). Consequently, the probability of RSV infection for individuals of age $a = 0$ is the one described by equation (1) in the main text. In contrast, the probability of RSV infection for an individual of age $a \geq 1$ in season $y$ becomes:

$$P_{a,y} = c_{mAbs}(y-a)\left(1 - e^{-s\lambda(a,y)}\right) + \left(1 - c_{mAbs}(y-a)\right)\left(P^1_{a,y}(0) + P^2_{a,y}(0)\right) \quad (13)$$

where $P^1_{a,y}(0)$ and $P^2_{a,y}(0)$ are defined as in the main analysis.

In the second sensitivity analysis (SA2), we consider a persisting protection against hospitalisation in the subsequent seasons following mAbs administration, in addition to the same reduction of susceptibility assumed in SA1.

However, SA2 differs from SA1 by the computation of the expected number of hospitalisations for individuals who received mAbs. The average joint probability of an individual in age-group $g$ who received mAbs in the past to get infected and hospitalised in season $y$ becomes:

$$\pi^{y,Hosp}_{g,mAbs} = \rho^{Hosp}_g (1 + \delta_a (r^{Hosp}_{mAbs} - 1)) \frac{\sum_{a \in g} P_{a,y}(\varepsilon_{mAbs}) N^y_a}{\sum_{a \in g} N^y_a} \quad (14)$$

where $r^{Hosp}_{mAbs}$ is the reduction in the hospitalisation risk among individuals who received mAbs in the past and who get infected in any season following the one of mAbs administration, and $\delta_a$ is defined as $\delta_a = 0$ when $a = 0$, and $\delta_a = 1$ otherwise.

To compute $r^{Hosp}_{mAbs}$, we approximate $P_{a,y}(\varepsilon_{mAbs}) \approx c_{mAbs}(y-a) \cdot s \cdot \lambda(a,y)$ and $P_{a,y}(\varepsilon_{mAbs}) \approx (1 - c_{mAbs}(y-a)) \cdot \lambda(a,y)$ for an individual of age $a > 0$. Since clinical trials estimated similar efficacy values against medically attended RSV-associated LRTI and against hospitalisation for RSV-associated LRTI [5], we can write:

$$P(hosp|mAbs) = (1 - \varepsilon_{mAbs}) P(hosp|no\ mAbs) \quad (15)$$

where $P(hosp|mAbs)$ and $P(hosp|no\ mAbs)$ denote the probability of being hospitalised for individuals who did and did not receive mAbs. Substituting the approximations for the probability of infection for an individual of age $a > 0$, equation (15) leads to:

$$r^{Hosp}_{mAbs} \rho^{Hosp}_a s \lambda(a,y) = (1 - \varepsilon_{mAbs}) \rho^{Hosp}_a \lambda(a,y) \implies r^{Hosp}_{mAbs} = \frac{1 - \varepsilon_{mAbs}}{s} \quad (16)$$

## Modelling the effect of mAbs and maternal vaccine

In our main analysis, we assume that the immunising intervention provides complete protection to a fraction of the individuals who received the treatment and no protection to

the remaining part of the treated population. In this case, the proportion of individuals receiving full protection by the intervention is thus computed as the coverage times the efficacy of the intervention. We conduct a sensitivity where we assume that all immunised individuals are exposed to a reduced risk of infection led by mAbs and maternal vaccine administration ('leaky' approach). In this case, considering one single intervention $int$, the probability of primary infection in season $y$ among individuals eligible for immunisation becomes:

$$P_{0,y}^{1}(r_{int}) = 1 - e^{-(1-r_{int})\cdot\lambda(0,y,r_{int})} \quad (17)$$

where $r_{int}$ is the reduction in the risk of infection led by the intervention and $\lambda(0,y,r_{int})$ is:

$$\lambda(0,y,r_{int}) = \beta(0,y)\cdot i(y)\cdot[x(0,y-1)\cdot c_{int}(y)(1-r_{int}) + 1 - x(0,y-1)c_{int}(y)] \quad (18)$$

We conducted two sensitivity analyses on the 'leaky' approach (L1 and L2), exploring two different possibilities for determining the value of $r_{int}$.

In the first analysis (L1), we consider how the estimated efficacy of nirsevimab was obtained in the clinical trials [5,6]:

$$\varepsilon_{mAbs} = 1 - RR = 1 - \frac{IC/(IC+IN)}{\bar{I}C/(\bar{I}C+\bar{I}N)} \quad (19)$$

where $RR$ is the relative risk, defined as the proportion of cases ($C$) occurred among the immunised ($I$) individuals divided by the proportion of cases occurred among non-immunised ($\bar{I}$) individuals ($N$ is the number of individuals who were not infected and reported).

For each season $y$, we can compute these quantities by applying the model as follows:

- $IC = N_0^y \cdot \rho_{0-4}^{Surv} \cdot c_{mAbs} \cdot (1 - e^{-(1-r_{mAbs})\cdot\lambda(0,y,r_{mAbs})})$
- $IN = N_0^y \cdot \rho_{0-4}^{Surv} \cdot c_{mAbs} \cdot e^{-(1-r_{mAbs})\cdot\lambda(0,y,r_{mAbs})}$
- $\bar{I}C = N_0^y \cdot \rho_{0-4}^{Surv} \cdot (1 - c_{mAbs}) \cdot \left(1 - e^{-\lambda(0,y,r_{mAbs})}\right)$
- $\bar{I}N = N_0^y \cdot \rho_{0-4}^{Surv} \cdot (1 - c_{mAbs}) \cdot e^{-\lambda(0,y,r_{mAbs})}$

Substituting these values in equation (19), we obtain:

$$\varepsilon_{mAbs} = 1 - \frac{IC/(IC+IN)}{\bar{I}C/(\bar{I}C+\bar{I}N)}$$

$$= 1 - \frac{\dfrac{N_0^y \cdot \rho_{0-4}^{Surv} \cdot c_{mAbs} \cdot \left(1 - e^{-(1-r_{mAbs})\cdot\lambda(0,y,r_{mAbs})}\right)}{N_0^y \cdot \rho_{0-4}^{Surv} \cdot [c_{mAbs} \cdot (1 - e^{-(1-r_{mAbs})\cdot\lambda(0,y,r_{mAbs})}) + c_{mAbs} \cdot e^{-(1-r_{mAbs})\cdot\lambda(0,y,r_{mAbs})}]}}{\dfrac{N_0^y \cdot \rho_{0-4}^{Surv} \cdot (1 - c_{mAbs}) \cdot (1 - e^{-\lambda(0,y,r_{mAbs})})}{N_0^y \cdot \rho_{0-4}^{Surv} \cdot [(1 - c_{mAbs}) \cdot (1 - e^{-\lambda(0,y,r_{mAbs})}) + (1 - c_{mAbs}) \cdot e^{-\lambda(0,y,r_{mAbs})}]}} \quad (20)$$

$$= 1 - \frac{1 - e^{-(1-r_{mAbs})\cdot\lambda(0,y,r_{mAbs})}}{1 - e^{-\lambda(0,y,r_{mAbs})}}$$

Rearranging equation (20) and recalling the explicit formulation of $\lambda(0, y, r_{mAbs})$, we can compute $r_{mAbs}$ for each season $y$ as the solution of the following equation:

$$\varepsilon_{mAbs} = e^{-\beta(0,y)\cdot i(y)\cdot[x(0,y-1)\cdot c_{mAbs}(y)(1-r_{mAbs})+1-x(0,y-1)\cdot c_{mAbs}(y)](1-r_{mAbs})} - (1-\varepsilon_{mAbs})\cdot e^{-\beta(0,y)\cdot i(y)\cdot[x(0,y-1)\cdot c_{mAbs}(y)(1-r_{mAbs})+1-x(0,y-1)\cdot c_{mAbs}(y)]} \quad (21)$$

Solving equation (21) requires numerical methods, as it is not solvable analytically. In our analyses, we used 'uniroot' function from R package stats (software version 4.1.2) to compute $r_{mAbs}$.

Efficacy values for RSVpreF vaccine were computed in the trials [7,8] as:

$$\varepsilon_{vax} = 1 - \frac{P}{1-P} = 1 - \frac{IC/(IC+\bar{I}C)}{1-IC/(IC+\bar{I}C)} \quad (22)$$

where $P$ is the proportion of cases among the individuals immunised by the maternal vaccine. Recalling the model derivation for $IC$and $\bar{I}C$, we get:

$$\begin{aligned}\varepsilon_{vax} &= 1 - \frac{IC/(IC+\bar{I}C)}{1-IC/(IC+\bar{I}C)} = 1 - \frac{IC/(IC+\bar{I}C)}{\bar{I}C/(IC+\bar{I}C)} = 1 - \frac{IC}{\bar{I}C} \\ &= 1 - \frac{N_0^y \cdot \rho_{0-4}^{Surv} \cdot c_{vax} \cdot \left(1 - e^{-(1-r_{vax})\cdot\lambda(0,y,r_{vax})}\right)}{N_0^y \cdot \rho_{0-4}^{Surv} \cdot (1-c_{vax}) \cdot (1 - e^{-\lambda(0,y,r_{vax})})} \\ &= 1 - \frac{c_{vax} \cdot \left(1 - e^{-(1-r_{vax})\cdot\lambda(0,y,r_{vax})}\right)}{(1-c_{vax}) \cdot (1 - e^{-\lambda(0,y,r_{vax})})}\end{aligned} \quad (23)$$

Since in the clinical trials the RSVpreF vaccine and the placebo were distributed in a 1:1 ratio [7,8], we can assume $c_{vax} = 1 - c_{vax}$. Under this assumption, equation (23) leads to an equation analogous to equation (21) obtained for mAbs. $r_{vax}$ can therefore be computed for each season $y$ by the numerical resolution of equation (21).

A further sensitivity analysis on the 'leaky' approach (L2) was conducted, simply assuming $r_{mAbs} = \varepsilon_{mAbs}$ and $r_{vax} = \varepsilon_{vax}$.

# Additional results and analyses

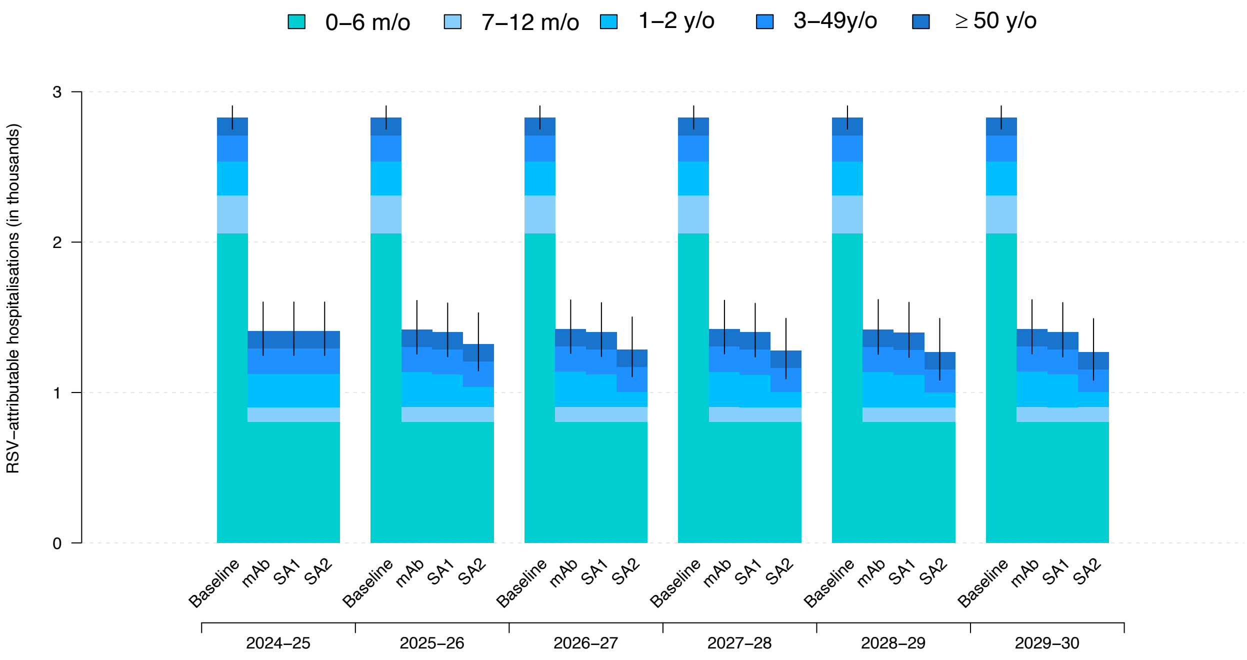


**Figure S5. Potential effect of routine seasonal mAbs administration on RSV-attributable hospitalisations.** Estimated number of hospitalisations due to RSV in Lombardy following annual mAbs administration from season 2024-25 to season 2029-30, as obtained in our main analysis and in the sensitivity analyses SA1 and SA2 when considering mAb administration to 80% of infants ("mAb") along with the corresponding estimates obtained under the no intervention scenario ("baseline"). Bars: mean estimates; lines: 95% CI.

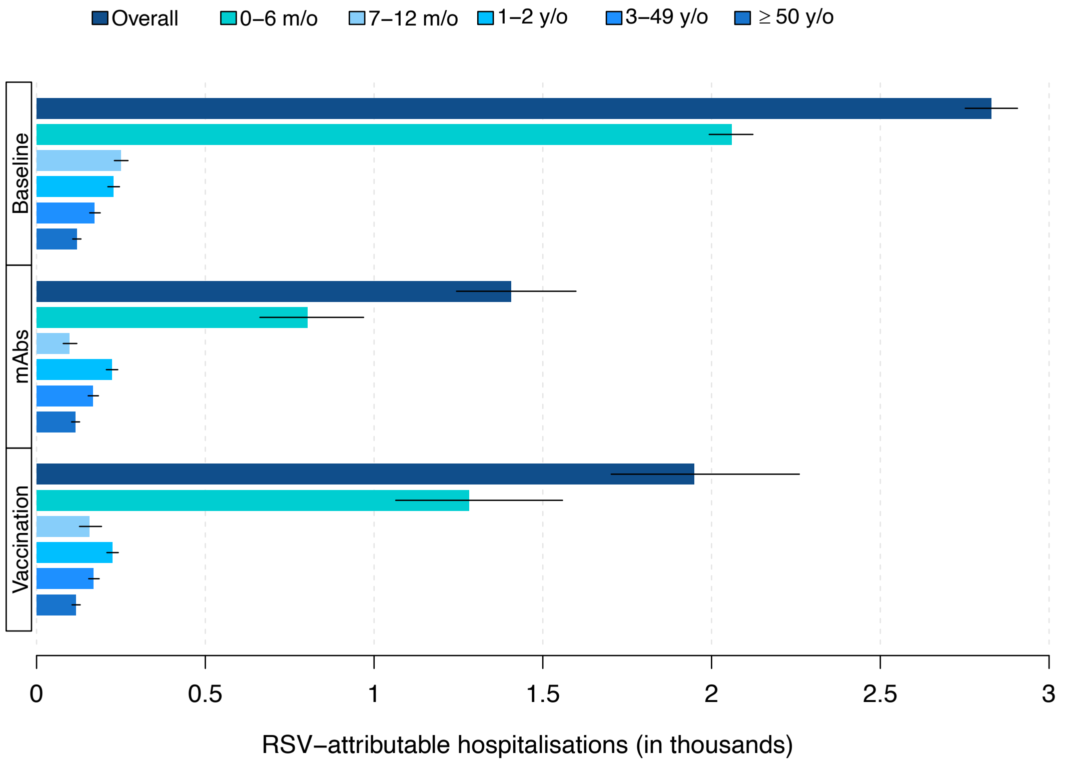


**Figure S6. Impact of different immunisation strategies on the age-stratified incidence of RSV-attributable hospitalisations in Lombardy, Italy for the season 2024-25, as obtained when assuming 'leaky' intervention (L1).** Model estimates of the age-specific number of RSV-attributable hospitalisations for 2024-2025 season when assuming no interventions ('baseline' scenario), mAbs administration to 80% of infants ('mAbs'), and vaccination of 65% of pregnant women ('vaccination').

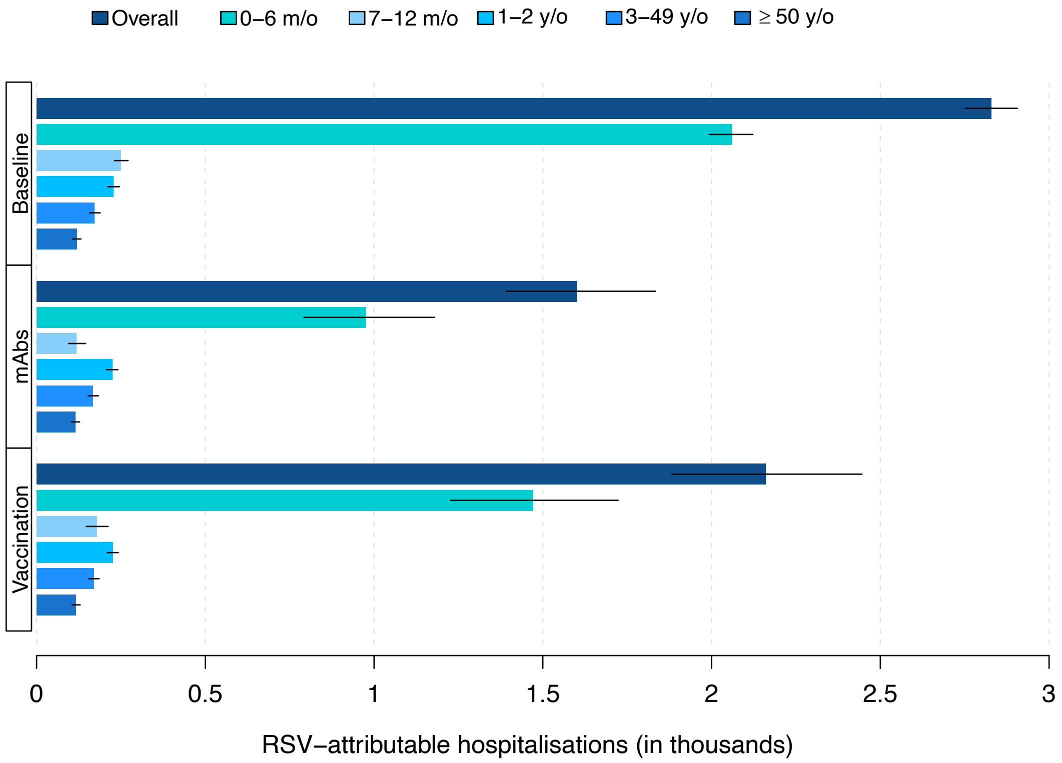


**Figure S7. Impact of different immunisation strategies on the age-stratified incidence of RSV-attributable hospitalisations in Lombardy, Italy for the season 2024-25, as obtained when assuming 'leaky' intervention (L2).** Model estimates of the age-specific number of RSV-attributable hospitalisations for 2024-2025 season when assuming no interventions ('baseline' scenario), mAbs administration to 80% of infants ('mAbs'), and vaccination of 65% of pregnant women ('vaccination').

**Table S2.** Numbers of averted cases and hospitalisations among infants following the introduction of nirsevimab administration in infancy or vaccination with RSVpreF vaccine during pregnancy in season 2024-25.

| Scenario | Efficacy | | Coverage | | Averted cases [95%CI] | % of averted cases [95%CI] | Averted hosp. [95%CI] | % of averted hosp. [95%CI] |
|---|---|---|---|---|---|---|---|---|
| | *mAbs* | *vaccine* | *mAbs* | *vaccine* | | | | |
| **mAbs** | 62%-85% | - | 80% | - | 5,975 [5,177 – 6,639] | 60.96 [52.91 - 67.76] | 1,408 [1,214 – 1,571] | 60.96 [52.91 - 67.76] |
| | | | 70% | - | 5,239 [4,537 – 5,823] | 53.46 [46.37 - 59.44] | 1,234 [1,064 – 1,378] | 53.46 [46.37 - 59.44] |
| | | | 95% | - | 7,072 [6,132 – 7,857] | 72.16 [62.66 - 80.18] | 1,667 [1,438 – 1,859] | 72.16 [62.66 - 80.18] |
| **Vaccination** | - | 30%-75% | - | - | 3,627 [2,326 – 4,729] | 37.01 [23.78 - 48.23] | 855 [549 – 1,113] | 37.01 [23.78 - 48.23] |

| | | | | | | | | |
|---|---|---|---|---|---|---|---|---|
| | | | - | - | 675 [431 - 883] | 6.89 [4.41 - 9] | 159 [102 - 208] | 6.89 [4.41 - 9] |
| | | | 65% | 57% | 4,453 [2,859 – 5,802] | 45.44 [29.23 - 59.18] | 1,049 [675 – 1,366] | 45.44 [29.23 - 59.18] |
| **Combined** | 62%-85% | 30%-75% | 80%* | 65% | 5,699 [4,382 – 6,817] | 58.15 [44.81 - 69.57] | 1,343 [1,035 – 1,609] | 58.15 [44.81 - 69.57] |
| | | | 70%* | 12% | 5,271 [4,594 – 5,848] | 53.79 [46.87 - 59.67] | 1,242 [1,079 – 1,384] | 53.79 [46.87 - 59.67] |
| | | | 95%* | 80% | 5,857 [4,249 – 7,202] | 59.76 [43.33 - 73.38] | 1,380 [1,002 – 1,698] | 59.76 [43.33 - 73.38] |

* These percentages are applied to the children whose mothers did not receive the vaccine during pregnancy

# References


1. Ang HJ, Menegale F, Preziosi G, Pariani E, Migliari M, Pellegrinelli L, et al. Reconstructing the impact of COVID-19 on the immunity gap and transmission of respiratory syncytial virus in Lombardy, Italy. EBioMedicine. 2023 Sep;95:104745.
2. Italian National Institute of Health (Istituto Superiore di Sanità). RespiVirNet - Sistema di Sorveglianza Integrata epidemiologica e virologica (Integrated epidemiological and virological surveillance system). [Accessed 2024 Sep 2]. Available from: https://www.epicentro.iss.it/influenza/respivirnet
3. Italian National Institute of Statistics (Istat). Lombardia population. Resident population on 1st January; 2022. [Accessed 2024 Jul 23]. Available from: http://dati.istat.it/?lang=en
4. Italian Ministry of Health (Ministero della Salute). Assistenza ospedaliera e dati SDO (Hospital discharge records). [Accessed 2024 Sep 4]. Available from: https://www.salute.gov.it/portale/assistenzaOspedaliera/homeAssistenzaOspedaliera.jsp
5. Muller WJ, Madhi SA, Seoane Nuñez B, Baca Cots M, Bosheva M, Dagan R, et al. Nirsevimab for Prevention of RSV in Term and Late-Preterm Infants. N Engl J Med. 2023 Apr 20;388(16):1533–4.
6. Hammitt LL, Dagan R, Yuan Y, Baca Cots M, Bosheva M, Madhi SA, et al. Nirsevimab for Prevention of RSV in Healthy Late-Preterm and Term Infants. N Engl J Med. 2022 Mar 3;386(9):837–46.
7. Kampmann B, Madhi SA, Munjal I, Simões EAF, Pahud BA, Llapur C, et al. Bivalent Prefusion F Vaccine in Pregnancy to Prevent RSV Illness in Infants. N Engl J Med. 2023 Apr 20;388(16):1451–64.
8. Fleming-Dutra KE, Jones JM, Roper LE, Prill MM, Ortega-Sanchez IR, Moulia DL, et al. Use of the Pfizer Respiratory Syncytial Virus Vaccine During Pregnancy for the Prevention of Respiratory Syncytial Virus–Associated Lower Respiratory Tract Disease in Infants: Recommendations of the Advisory Committee on Immunization Practices — United States, 2023. MMWR Morb Mortal Wkly Rep. 2023 Oct 13;72(41):1115–22.
